# Successful use of daily intravenous infusion of C1 esterase inhibitor concentrate in the treatment of a hereditary angioedema patient with ascites, hypovolemic shock, sepsis, renal and respiratory failure

**DOI:** 10.1186/s13223-014-0062-9

**Published:** 2014-12-11

**Authors:** Hoang Pham, Stephanie Santucci, William H Yang

**Affiliations:** Faculty of Medicine, University of Ottawa, Ottawa, Ontario Canada; Allergy & Asthma Research Centre, Ottawa, Ontario Canada

**Keywords:** Berinert®, Hereditary angioedema, HAE, C1 inhibitor concentrate, C1-INH, Short-term prophylaxis, Critical care

## Abstract

Hereditary angioedema (HAE) is a rare autosomal dominant disease most commonly associated with defects in C1 esterase inhibitor (C1-INH). HAE manifests as recurrent episodes of edema in various body locations. Atypical symptoms, such as ascites, acute respiratory distress syndrome, and hypovolemic shock, have also been reported. Management of HAE conventionally involves the treatment of acute attacks, as well as short- and long-term prophylaxis. Since attacks can be triggered by several factors, including stress and physical trauma, prophylactic therapy is recommended for patients undergoing surgery. Human plasma-derived C1-INH (pdC1-INH) concentrate is indicated for the treatment of both acute HAE attacks and pre-procedure prevention of HAE episodes in patients undergoing medical, dental, or surgical procedures. We report the first case of a patient with HAE who experienced an abdominal attack precipitated by a retroperitoneal bleed while being converted from warfarin to heparin in preparation for surgery. Subsequently, the patient had a protracted course in hospital with other complications, which included hypovolemic shock, ascites, severe sepsis from nosocomial pneumonia, renal and respiratory failure. Despite intensive interventions, the patient remained in a critical state for months; however, after a trial of daily intravenous infusion of pdC1-INH concentrate (Berinert®, CSL Behring GmbH, Marburg, Germany), clinical status improved, particularly renal function. Therefore, pdC1-INH concentrate may be an effective treatment option to consider for critically-ill patients with HAE.

## Background

Hereditary angioedema (HAE) is an autosomal dominant genetic disease affecting approximately 1 in 50,000 people worldwide, with no ethnic differences reported [1]. Recently, a new classification system has been described for angioedema and this case report focuses on hereditary angioedema with C1 esterase inhibitor (C1-INH) deficiency [2]. This rare disease is due to a mutation in one of the alleles of the C1-INH gene, SERPING1, which results in reduced plasma levels of C1-INH and dysregulation of the contact system, facilitating an increased release of bradykinin, the key mediator of angioedema.

C1-INH plays an important role in downregulating several interconnected pathways including the complement, contact, fibrinolytic, and coagulation systems [3]. Activation of these systems whether by trauma, infection, febrile illness, or other unknown factors consumes C1-INH as it is a ‘suicide inhibitor’ [4].

HAE is characterized by recurrent episodes of localized non-pruritic edema, painful abdominal attacks, and angioedema of the upper airway [5]. Laryngeal swelling poses the greatest risk to patient health, and can lead to death from upper airway obstruction. Attacks can be triggered by several factors, including stress, physical trauma, and infection, but also can occur spontaneously. In many cases, attacks are preceded by prodromal symptoms such as fatigue, irritability, weakness, nausea, and erythema marginatum [6]. Swelling sometimes occurs rapidly, but typically worsens gradually over the first 24 hours. Attacks can last for 1–5 days, and usually resolve over 48–72 hours [5,7]. The frequency of attacks, sites of edema, and precipitating events can vary widely, both between individuals and within the same patient [5,7].

In addition to the common clinical presentations described above, there have been case reports of HAE being associated with some severe conditions: acute respiratory distress syndrome (ARDS) [8], ascites [9-14], and hypovolemic shock [15]. The pathophysiology of these associations has not been adequately elucidated yet.

Management of HAE involves the treatment of acute attacks, as well as short- or long-term prophylaxis. Several effective HAE treatments are available, and various international guidelines and recommendations have been published to inform their optimum use [1,6,16,17]. However, low awareness of HAE often leads to delayed, suboptimal, or lack of treatment. This can result in unnecessary morbidity and, in some instances, fatality.

Furthermore, as HAE is a systemic disease, the treatment of comorbid conditions and routine medical procedures can pose a problem, which may require intervention to prevent attacks [17,18]. Surgery is a particular concern for patients with HAE, as retrospective studies have shown an increased risk of upper airway angioedema complications, especially with oral surgeries [19]. Several agents are recommended for short-term prophylaxis to prevent attacks during major surgery, though plasma-derived CI-INH (pdC1-INH) concentrates (Berinert®, CSL Behring GmbH, Marburg, Germany and Cinryze®, ViroPharma, Exton PA, USA and ViroPharma Europe, Brussels, Belgium) are generally the favored options [17]. The 2012 World Allergy Organization recommended dose of intravenous (IV) pdC1-INH for acute attack is 20 units/kg (Berinert®) or 1000 units (Cinryze®) [17]. For short-term prophylaxis, guideline suggestions vary between 10-20 units/kg or 1000 units, 1–6 hours before the procedure [17]. Alternatively, attenuated androgens can also be used for short-term/pre-procedural prophylaxis for 5 days before and 2–5 days after the event with danazol at 2.5–10 mg/kg/day (maximum of 600 mg) or stanozolol at 4–6 mg/day [17].

Berinert® (CSL Behring GmbH, Marburg, Germany) is a highly purified pdC1-INH concentrate that treats the root cause of HAE symptoms by replacing the deficient or dysfunctional C1-INH. In Europe, pdC1-INH concentrate is indicated for the treatment of both acute HAE attacks and pre-procedure prevention of acute episodes of HAE in adult and pediatric patients undergoing medical, dental, or surgical procedures [20]. The efficacy and safety of pdC1-INH concentrate for the long-term treatment of successive HAE attacks at different anatomical sites has been demonstrated in numerous observational and descriptive studies [21].

Here we describe the use of pdC1-INH concentrate (Berinert®) in a patient with HAE with comorbidities who developed major complications prior to scheduled heart surgery, including retroperitoneal bleed, hypovolemic shock, ascites, severe sepsis from nosocomial pneumonia, renal and respiratory failure.

## Case presentation

### Patient history

This case report involves a 51-year-old man with type I HAE, who first developed symptoms in his late teens. No genetic testing had been carried out, but laboratory tests showed a C1-INH level of 73 mg/L and a C4 level of <0.10 g/L, which are both below the normal ranges of 210–350 mg/L and 0.16–0.47 g/L, respectively. In addition to HAE, the patient also suffered from the neurological disorder Charcot-Marie-Tooth disease, and congenital aortic stenosis. His sister also had both HAE (age of onset also in late teens) and Charcot-Marie-Tooth disease [22]. The patient was experiencing frequent attacks of abdominal HAE, occurring approximately every 4–6 weeks.

### Surgical history

In 1997, the patient underwent successful open-heart surgery (aortic stenosis repair). In preparation for the surgery, the patient was receiving oral danazol (200 mg, three times a day). He was also given one dose of 1500 units IV pdC1-INH concentrate preoperatively (his weight was approximately 78 kg). The patient was satisfied with the outcome and his quality of life was substantially improved.

In 2000, the patient required emergency surgery due to an extensive ascending aorta aneurysm dissection. Surgery lasting 12 hours was carried out to replace the ascending aorta. At the time of operation, he was receiving oral stanozolol (4 mg, four times a day), as the surgeon chose to prescribe stanozolol over danazol. This higher than normal dosing was only prescribed for 1 week in preparation for the emergency surgery (4–6 mg/day is the norm). Stanozolol was reduced to 12 mg daily after about 1 month and then tapered down to 4 mg daily shortly after that. The operation had a successful outcome. Postoperatively, he had an abdominal HAE attack and was treated with 1500 units of IV pdC1-INH concentrate.

### Surgical complications

In 2004, the patient was admitted for a scheduled aortic aneurysm repair. He was receiving oral danazol (200 mg, twice daily) as stanozolol had been removed from the market at this time. However, the procedure was cancelled as complications arose 3 days before the scheduled surgery; hence, no prophylactic C1-INH had been provided.

In preparation for the surgery, the patient’s anticoagulation was bridged from long-term warfarin to heparin. Three days into his heparin protocol, the patient complained of left flank pain and severe abdominal pain. On physical examination, the patient was agitated, disoriented, pale, and peripherally cold. His vital signs consisted of a temperature (temp) of 35.2°C, heart rate (HR) of 91 beats per minutes (bpm), blood pressure (BP) of 70/40 mm Hg, and respiratory rate (RR) of 26 breaths/min. Laboratory results revealed low hemoglobin at 43 g/L (was 118 g/L 2 days prior), low hematocrit of 0.24, leukocytosis at 22 × 10^9^/L, hyperglycemia at 8.6 mmol/L, and acute renal failure with creatinine (Cr) at 290 umol/L (up from 64 umol/L 5 days earlier) and zero urine output. Resuscitation interventions included discontinuation of heparin, sedation with propofol, intubation, mechanical ventilation (FiO_2_ = 0.40, PEEP = 10), and renal replacement therapy (RRT). Red blood cells, IV fluids, norepinephrine (vasopressor), and pdC1-INH concentrate (2000 units) were also administered. An abdominal computed tomography (CT) scan showed a massive retroperitoneal hemorrhage, slight intraperitoneal bleeding, and moderate ascites. Ascites was clinically confirmed the following morning as the patient had asymmetric abdomen distention and tautness in the right and left upper quadrants with rebound tenderness and guarding in the right lower quadrant. His abdominal girth at this time was 106 cm. The care team had the clinical impression that the patient had an HAE abdominal crisis precipitated by a spontaneous retroperitoneal bleed followed by the development of ascites, hypovolemic shock, and acute renal failure. Repeat CT scan revealed the retroperitoneal hematoma was stable, organized, and contained.

Two days later, the patient contracted hospital-acquired pneumonia (subsequently shown to be caused by *Klebsiella pneumoniae*, *Staphylococcus aureus,* and *Pseudomonas aeruginosa*).

A chest x-ray (CXR) showed extensive bilateral pneumonia. The patient was treated with different antibiotics (ciprofloxacin, piperacillin/tazobactam, vancomycin, metronidazole, cloxacillin, rifampin, and tobramycin) over the next few weeks in attempts to clear up the bacterial infection.

Simultaneously with regard to renal function, the patient was diagnosed with ischemic acute tubular necrosis. Renal function improved for a short time following the initial RRT with Cr trending down to 124 umol/L, but 7 days after the initial bleed, renal function deteriorated again with Cr rising to 139 umol/L, most likely due to increasing sepsis as the patient developed a temp of 40.4°C, HR of 138 bpm, BP of 115/50 mmHg, RR of 27 breaths/min, and leukocytosis of 30.3 × 10^9^/L. This second round of acute kidney injury necessitated reinitiating RRT 2 days later, as Cr reached 322 umol/L. Three days later, the patient’s ascites, which had begun to resolve, started to increase again in the absence of new bleed (according to a CT scan). The next day, dialysis became clinically indicated. In addition to worsening kidney function, the patient showed signs of hypoxemic respiratory failure with mild oxygen desaturation (93–97%) despite being on a bi-level ventilator. Two days later, the respiratory failure required a tracheotomy for assisted ventilation. His active pneumonia was implicated as the suspected source of the severe sepsis. However, the care team’s differential diagnosis for the hypoxemic respiratory failure consisted of extensive nosocomial pneumonia, severe sepsis from the pneumonia, ARDS, or a combination of the three. Discerning between the three processes was a challenge because they were highly interrelated. Severe pneumonia and sepsis can cause ARDS while severe sepsis and ARDS can lead to multisystem dysfunction. Furthermore, severe pneumonia and ARDS share similar symptoms, signs, and radiographic findings (i.e. the patient’s CXR showed bilateral patchy infiltrates which could be due either to pneumonia or alveolar pulmonary edema). Over the course of the following weeks, the patient continued to experience renal failure requiring dialysis up to 4 times a week.

Despite 2 months of optimal intensive care unit (ICU) interventions including renal dialysis, assisted ventilation, antibiotic therapy, blood transfusions, daily danazol (200 mg), and very conservative intermittent pdC1-INH concentrate, the patient remained in critical condition and the decision to terminate his life was considered. Given a lack of clinical and hemodynamic response to ongoing conventional treatment, a decision was made to administer daily pdC1-INH concentrate as a trial to see if the multi-organ failure and ascites would be alleviated with better control the patient’s HAE via C1-INH replacement. An IV infusion of pdC1-INH concentrate (1000 units) was administered for 21 days (total: 21000 units).

Within 48 hours of daily pdC1-INH concentrate infusion, renal function improved (improved urine output and stabilization of Cr levels). After 4 more days, Cr values started decreasing. Eventually, abdominal ascites and pneumonia clinically resolved as well. The care team successfully weaned him off the respirator. The patient regained his full strength after further reconditioning and returned home 4 months after being admitted for aortic aneurysm repair.

## Discussion and conclusions

Given the role of C1-INH in various pathways and tremendous physiologic stress of this patient’s lengthy stay in the ICU (involving complications such as retroperitoneal hemorrhage, hypovolemic shock, ascites, renal failure, nosocomial pneumonia, severe sepsis, and hypoxemic respiratory failure), it is likely that HAE was inadequately controlled, resulting in multiple relapses in this context of critical illness. Treatment with daily infusions of pdC1-INH concentrate optimized control of HAE.

Although the anti-inflammatory role of C1-INH therapy has been explored mostly in animal studies, there have been some studies in which the therapeutic administration of pdC1-INH concentrate may have beneficial effects on patients in a variety of clinical scenarios, even non-HAE patients. These include conditions such as sepsis, ischemia-reperfusion injury, emergency coronary artery bypass grafting, transplantation [23]; septic shock, vascular leak syndrome, severe thermal injury [24]; and toxic shock syndrome [25]. In a double blind, randomized placebo-controlled trial of 40 patients with severe sepsis and septic shock, investigators infused patients over 1 hour with pdC1-INH concentrate IV, starting with 6000 units, followed by 3000, 2000, and 1000 units (total: 12000 units) at 12-hour intervals. Patients treated with pdC1-INH concentrate had less severe multi-organ dysfunction and significantly improved renal function compared with patients treated with placebo [26]. In the same group of patients, researchers found that neutrophil activation, which is elevated in sepsis and correlated with severity of organ dysfunction, was reduced by C1-INH therapy. This could partially account for the mildly beneficial treatment effects on organ dysfunction in sepsis [27]. With respect to survival, pdC1-INH concentrate infusion of 12000 units (6000 units/day for 2 days) increased survival rates for patients with sepsis through downregulation of the systemic inflammatory response [28]. In terms of renal function, the investigators were uncertain as to whether C1-INH improved kidney function through local inhibition of complement and contact activation or by prevention of extensive capillary leakage [26].

In a previous report, a young woman with Caroli’s disease suffering from septic shock with capillary leakage syndrome, had received ultimate rescue therapy consisting of 60 units/kg pdC1-INH concentrate infusion over 1 hour followed by continuous administration of 30 units/kg pdC1-INH concentrate per day for 4 days. Her hemodynamics rapidly stabilized with a reduction in vasopressor medication and normalization of fluid balance [29].

In another small clinical trial of seven patients with streptococcal toxic shock syndrome (septic shock with multi-organ failure), all patients received 2–3 high doses of pdC1-INH totaling 6000–10000 units within the first 24 hours after admission. A marked shift of fluid from the extravascular to intravascular compartment was observed (in all but one patient); leading to transient pulmonary edema but also a rapid decrease in the need for vasopressors and fluid replacement [25].

Thus, this literature, whereby even non-HAE patients gained treatment benefits from C1-INH therapy in a number of critical conditions, helps generate hypotheses to explain some of the treatment benefits of daily infusion of pdC1-INH observed in this case report patient. Furthermore, the long-standing history of HAE in this patient and the fact that he had benefited from the use of pdC1-INH infusion during the previous two major surgical interventions in 1997 and 2000, provided further support that C1-INH infusion likely facilitates much greater control of HAE.

In summary, here we report the first case of HAE with pre-surgical multi-organ failure and significant ascites, likely due to an abdominal HAE attack. The patient responded to IV infusion of pdC1-INH concentrate (1000 units), administered daily for 21 days. Daily IV infusion of pdC1-INH concentrate, in addition to conventional supportive measures, may be an effective option to consider in preventing complications in critically ill patients with HAE. As shown in this case report, HAE can be potentially life-threatening. It is important for patient/family and treating physicians to be aware of this outcome, in order to prevent future occurrences. Lack of awareness of HAE may have contributed to a delay in treatment and unnecessary suffering. Furthermore, the outcome in this case would most likely have been fatal without pdC1-INH concentrate. This case highlights the need to raise the profile of HAE within the medical community.

## Consent

Written informed consent was obtained from the next of kin of the patient for publication of this case report and any accompanying images. A copy of the written consent is available for review by the Editor-in-Chief of this journal.

## Abbreviations

ARDS: Acute respiratory distress syndrome
BP: Blood pressure
bpm: Beats per minute
C1-INH: C1 esterase inhibitor
Cr: Creatinine
CT: Computed tomography
CXR: Chest x-ray
FiO_2_: Fraction of inspired oxygen
HAE: Hereditary angioedema
HR: Heart rate
ICU: Intensive care unit
IV: Intravenous
PEEP: Positive end-expiratory pressure
pdC1-INH: Plasma-derived C1-INH
RR: Respiratory rate
RRT: Renal replacement therapy
temp: Temperature
